# Progression of Brain Atrophy in Spinocerebellar Ataxia Type 2: A Longitudinal Tensor-Based Morphometry Study

**DOI:** 10.1371/journal.pone.0089410

**Published:** 2014-02-25

**Authors:** Mario Mascalchi, Stefano Diciotti, Marco Giannelli, Andrea Ginestroni, Andrea Soricelli, Emanuele Nicolai, Marco Aiello, Carlo Tessa, Lucia Galli, Maria Teresa Dotti, Silvia Piacentini, Elena Salvatore, Nicola Toschi

**Affiliations:** 1 Quantitative and Functional Neuroradiology Research Program at Meyer Children Hospital and Careggi General Hospital, Florence, Italy; 2 “Mario Serio” Department of Experimental and Clinical Biomedical Sciences, University of Florence, Florence, Italy; 3 Department of Electrical, Electronic, and Information Engineering “Guglielmo Marconi”, University of Bologna, Cesena, Italy; 4 Unit of Medical Physics, Pisa University Hospital “Azienda Ospedaliero-Universitaria Pisana”, Pisa, Italy; 5 Neuroradiology Unit, Careggi General Hospital, Florence, Italy; 6 IRCSS SDN Foundation, Naples, Italy; 7 University of Naples Parthenope, Naples, Italy; 8 Unit of Radiology, Versilia Hospital, Azienda USL 12 Viareggio, Lido di Camaiore (Lu), Italy; 9 Molecular Medicine Unit, Le Scotte University Hospital, Siena, Italy; 10 Department of Medicine, Surgery and Neuroscience, University of Siena, Siena, Italy; 11 Department NEUROFARBA, University of Florence, Florence, Italy; 12 Department of Neurological Sciences, University of Naples Federico II, Naples, Italy; 13 Medical Physics Section, Department of Biomedicine and Prevention, University of Rome “Tor Vergata”, Rome, Italy; 14 Department of Radiology, Athinoula A. Martinos Center for Biomedical Imaging, Boston, Massachusetts, United States of America; 15 Harvard Medical School, Boston, Massachusetts, United States of America; University of Manchester, United Kingdom

## Abstract

Spinocerebellar ataxia type 2 (SCA2) is the second most frequent autosomal dominant inherited ataxia worldwide. We investigated the capability of magnetic resonance imaging (MRI) to track *in vivo* progression of brain atrophy in SCA2 by examining twice 10 SCA2 patients (mean interval 3.6 years) and 16 age- and gender-matched healthy controls (mean interval 3.3 years) on the same 1.5 T MRI scanner. We used T1-weighted images and tensor-based morphometry (TBM) to investigate volume changes and the Inherited Ataxia Clinical Rating Scale to assess the clinical deficit. With respect to controls, SCA2 patients showed significant higher atrophy rates in the midbrain, including substantia nigra, basis pontis, middle cerebellar peduncles and posterior medulla corresponding to the gracilis and cuneatus tracts and nuclei, cerebellar white matter (WM) and cortical gray matter (GM) in the inferior portions of the cerebellar hemisphers. No differences in WM or GM volume loss were observed in the supratentorial compartment. TBM findings did not correlate with modifications of the neurological deficit. In conclusion, MRI volumetry using TBM is capable of demonstrating the progression of pontocerebellar atrophy in SCA2, supporting a possible role of MRI as biomarker in future trials.

## Introduction

Spinocerebellar ataxia type 2 (SCA2) is the second most frequent autosomal dominant inherited ataxia worldwide after SCA3 [1], [2]. SCA2 is a typical neurodegenerative disease, namely a progressive and ultimately fatal disorder [3]. Despite active research [3], neither effective drugs nor the therapeutic window to slow, halt and hopefully reverse the degenerative process in SCA2 have been established to date. In this context, identification of reliable and sensitive biomarkers of disease progression, which could potentially be employed in future trials, is extremely desirable.

Cross-sectional magnetic resonance imaging (MRI) studies using T1-weighted images have allowed *in vivo* detection of brainstem and cerebellar atrophy in SCA2, as well as the investigation of possible correlations between volume loss and genetic or clinical features [4]–[16]. So far, four longitudinal MRI studies have used T1-weighted imaging in conjunction with global volumetry or voxel-based morphometry (VBM) to investigate the progression of brain atrophy in autosomal dominant inherited ataxias (including SCA1, SCA3, SCA6 [17], [18] and SCA17 [19]) as well as in multi system atrophy (MSA) [20]. To the best of our knowledge, the longitudinal progression of atrophy in SCA2 has not yet been investigated through MRI.

Tensor-based morphometry (TBM) is an advanced approach to evaluating local volume differences and changes in cross-sectional and longitudinal studies, respectively. TBM is preferable to VBM analysis since it does not need to rely on preventive gray matter (GM) and white matter (WM) segmentation (which can be particularly cumbersome in the brainstem and cerebellum) [21]–[28]. It employs high-dimensional nonlinear deformations to either register every subject's anatomy to a custom-built, study-specific template (cross sectional studies) or to register each subject's late T1-weighted image to their early T1-weighted image (longitudinal studies), after which local volumetric differences or changes are assessed by computing the voxel-wise Jacobian determinant **|J|** of the deformation fields [21]–[31]. Subsequently, the time-normalized amount of local volume variation, sometimes referred to as yearly warp rate (WR), can be calculated. The advantages of TBM in longitudinal studies have recently been highlighted [24].

In the present study, we used high-resolution T1-weighted MRI acquisitions of the brain in conjunction with an optimized, *ad hoc* implementation of longitudinal TBM in order to track the progression of local atrophic changes over a mean time span of 3.4 years in 10 patients with SCA2 and 16 age- and gender-matched healthy controls. Additionally, we investigated possible correlations between longitudinal rates of volumetric change and clinical modifications over the same time span.

## Materials and Methods

### Participants

Ten patients (4 women, 6 men; mean age 47.5±12.7 years) with a genetically confirmed diagnosis of SCA2 [32] gave their written consent to participating in this longitudinal study. The study was approved by the Ethic Committee of Careggi University Hospital of Florence. The cut-off number of triplet repeats expansions qualifying for diagnosis of SCA2 was 34 CAG on one allele, and the mean number of abnormal triplets was 40.6±1.4 (Table 1).

**Table 1:** Demographic, genetic and clinical data in 10 SCA2 patients.

Patient #	Age (years)	Gender	Number of triplet repeats expansions	Disease duration (years)	IACRS baseline	IACRS follow-up
1	60.6	M	38	20	20	23
2	28.4	M	39	2	9	14
3	31.5	M	43	7	16	16
4	46.5	F	41	7	17	21
5	47.4	F	41	10	19	27
6	43.7	M	41	8	15	15
7	67.8	M	41	23	14	18
8	54.5	F	42	23	25	31
9	56.9	M	40	14	20	27
10	37.7	F	40	14	17	21
Mean (SD)	47.5 (12.7)		40.6 (1.4)	12.8 (7.3)	17.2 (4.3)	21.3 (5.7)

F, female; IACRS, Inherited Ataxia Clinical Rating Scale; M, male; SD, standard deviation.

Patients were examined twice using the same 1.5 T MRI scanner and acquisition protocol, on average 3.6±0.7 years apart (range 2.2-4.1). At the time of both MRI examinations, all patients also underwent evaluation by the same clinician (A.G.) who assessed disease duration since onset of symptoms and the neurological deficit using the Inherited Ataxia Clinical Rating Scale (IACRS) [33]. The IACRS is a 0–38 scale (where 38 corresponds to maximum clinical deficit), originally developed for Friedreich ataxia, which evaluates signs and symptoms related to ataxia but also to pyramidal tract dysfunction and impaired vibration or position sense. Due to this improved capability to rate the global neurological deficit observed in SCA2 patients (related to damage of the brainstem, cerebellum, and long motor and sensitive pathways) the IACRS was preferred to the Scale for the Assessment and Rating of Ataxia (SARA) which evaluates cerebellar symptoms and signs [12].

At the time of baseline MRI, the mean disease duration from clinical onset in SCA2 patients was 12.8±7.3 years (range 2–23) and the mean IACRS score was 17.2±4.3 (range 9–25) (Table 1).

A group of 16 age- and gender-matched healthy subjects (7 women, 9 men; mean age 50.3±18.8 years) were recruited as controls and gave written consent to participating in the study. They had no history of neurological or psychiatric dysfunction and their neurological examination was negative. Controls were examined twice, on average 3.3±1.0 years apart (range 1.9–4.7), using the same 1.5 T MRI scanner and acquisition protocol employed for SCA2 patients. Source T1 weighted images in patients and controls can be made freely available upon request. VBM results at baseline examination in 9 of our SCA2 patients and 10 out of the healthy controls have been previously reported [8].

### MRI examination

All patients and controls underwent examination in a single centre on a 1.5 T MRI scanner (Philips Intera, Best, The Netherlands) equipped with 33 mT/m maximum gradient strength and SENSE 6-channel coil technology. After the scout, sagittal 3D T1-weighted turbo gradient echo [repetition time (TR) = 8.1 ms, echo time (TE) = 3.7 ms, flip angle = 8°, inversion time = 764 ms, field of view (FOV) = 256 mm×256 mm, matrix size = 256×256, 160 contiguous slices, slice thickness = 1 mm] images were acquired for analysis through TBM.

### Data analyses

#### Preprocessing

T1-weighted images were visually evaluated by an expert neuroradiologist (M.M.) for potential motion artifacts before entering further image processing. After this visual quality check, all images were retained for further processing.

#### TBM

All registration procedures were based on variations of the SyN algorithm which, in a comparison of 14 registration strategies, has been shown to provide the most consistently high accuracy [34]. We employed the Greedy SyN implementation of the SyN algorithm included in the ANTs package [35]. All image registrations were initialized through a 12-degree of freedom (DOF) affine transform which was followed by a nonlinear diffeomorphic step, and employed neighborhood cross-correlation as similarity metric. For improved accuracy with respect to the default package settings, in the second (nonlinear) registration step we used four multiresolution levels with a maximum number of 200 iterations per level and smoothing resolutions of 3, 2, 1 and 0 mm per level (from coarsest to finest).

#### Custom T1 template construction

In order to generate an unbiased, population-specific T1 template, we used baseline T1-weighted images from all (number of subjects = 10) patients and an equal number of randomly selected controls. The template was generated using a procedure similar to that described in [36]. Briefly, after N4 bias field correction [37], an initial template was bootstrapped from all baseline images and coregistrations of individual brain images were iteratively refined to create a group average, which is often referred to as an optimal average template. In particular, the algorithm works within the diffeomorphic space towards building an average shape and appearance brain by reducing dependence on the topological idiosyncrasy of any individual brain. Within the ANTs package, the SyN tool is called to nonlinearly coregister all brain images to one another in an iterative manner for subsequent intensity averaging. The procedure is repeated recursively, thereby iteratively refining the co-registration of the constituent images. Five global iterations were used to build the final template in this study. Creation of the custom template required approximately 350 hours of CPU time.

#### Image registration

All N4-corrected baseline images were registered to the unbiased template as described above. The voxel-wise jacobian determinant of the nonlinear component of the warpfield (**|J|**_baseline_) was then computed for each subject. In particular, when the local volume in N4-corrected image is greater/less than local volume in the unbiased template image the **|J|**_baseline_ values are less/greater than 1, respectively.

Also, every N4-corrected follow-up image was registered (intra-subject) to the N4-corrected baseline image as described above, and the voxel-wise jacobian determinant **|J|**_longitudinal_ of the nonlinear component of the inverse warpfield (i.e. relative to the transformation which takes the baseline image into the space of the follow-up image) was computed (resulting in **|J|**_longitudinal_ being in baseline image space). The voxel-wise WR was then computed as WR = (**|J|**_longitudinal_ - 1)/*t*, where *t* is time (in years) between baseline and follow-up imaging. In particular, WR values less/greater than 0 indicate contraction/expansion of local tissue volume, respectively. Finally, maps of voxel-wise WR were transformed into custom T1 template space by applying the transformations computed in the previous step (which take baseline T1-weighted images into custom T1 template space).

#### Statistical analyses of atrophy rates

All voxel-wise statistical analyses were performed within the framework of the general linear model (GLM) while controlling for age and gender as nuisance covariates.

We employed non-parametric, permutation-based inference approach which included full correction for multiple comparisons over space. In particular, p-values were calculated and corrected for multiple comparisons using the “3D” parameter setting with threshold-free cluster enhancement (TFCE), thereby avoiding the use of an arbitrary amount of spatial smoothing as well as threshold for the initial cluster-formation, which can affect the sensitivity of statistical analysis and bias results [38]. For each comparison, we employed 50000 permutation for increased accuracy, and corrected p-values smaller than 0.05 were considered statistically significant. Results of all TFCE analyses were interpreted separately. The comparisons we performed were a) patients vs. controls at baseline (between-group cross-sectional analysis, quantity of interest: **|J|**_baseline_), b) follow-up vs. baseline (within-group longitudinal analysis and group x time interaction, quantity of interest: WR) and c) within-patient group correlation of WR with number of abnormal CAG triplets, disease duration and variations (follow-up-baseline) of the neurological deficit as assessed with IACRS scale. In a) we tested the effect of group, in b) we tested the effect of time (in each group) and of group x time interaction, in c) we tested the hypothesis of a regression slope being larger or smaller than zero. Resulting p-value maps were transformed into MNI-152 space by applying an affine, 12-DOF transformation computed by registering the custom T1 template to the MNI-152 template. In order to avoid creating false p-values, nearest neighbor interpolation was employed in this step.

## Results

**Figure 1:**
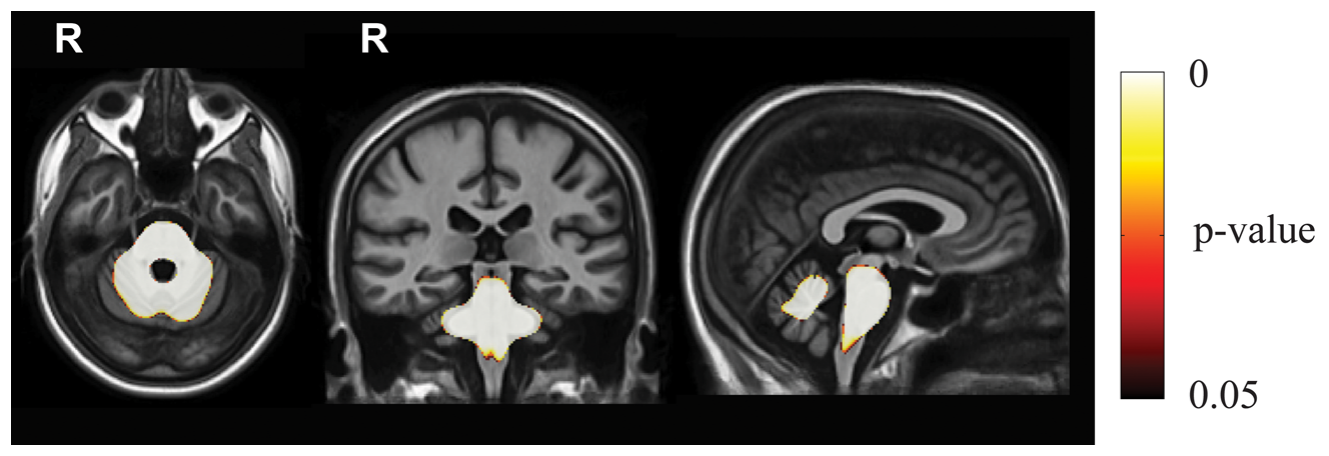
Results of the baseline between group (SCA2 vs. controls) TBM analysis. Voxel-wise corrected p-value maps (threshold-free cluster enhancement, TFCE), testing the null hypothesis of zero differences in **|J|**_baseline_ between SCA2 patients and healthy controls. Highlighted clusters indicate significantly (p<0.05) more pronounced mean atrophy in SCA2 patients when compared to healthy controls (i.e. **|J|**_baseline_ in SCA2 patients significantly lower than **|J|**_baseline_ in healthy controls). All maps are overlayed on population-specific T1 template. These maps show significant symmetric atrophic changes in SCA2 patients (with respect to controls) in the brainstem, middle cerebellar peduncels, and cerebellar WM and adjacent cortical GM. No significant differences are observed in the supratentorial compartment.

Between-group cross-sectional analysis at baseline showed significant symmetric atrophic changes in SCA2 patients (with respect to controls, i.e. the **|J|**_baseline_ in SCA2 patients were significantly lower than in controls) in the brainstem, middle cerebellar peduncels, cerebellar WM and adjacent cortical GM. No significant differences were found in the supratentorial compartment (Figure 1, Table S1).

Within-group longitudinal analysis in control subjects showed a circumscribed atrophy (i.e. WR values were significantly smaller than 0) of the right frontal mid-orbital gyrus and underlying WM (Figure S1, Table S2) and a symmetrical enlargement (i.e. WR values were significantly greater than 0) of the lateral ventricles.

Within-group longitudinal analysis in SCA2 patients (Figure S2, Table S3) showed, in the supratentorial compartment, volume loss (i.e. WR values were significantly smaller than 0) of the WM in the left frontal gyrus, temporal lobe, anterior limb of the internal capsule, and peritrigonal region, and of the GM in the left midfrontal gyrus, putamen and pallidus and in the thalamus and hippocampus, bilaterally. The third and lateral ventricles showed enlargement (i.e. WR values were significantly greater than 0). In the infratentorial compartment, diffuse volume loss was present in the midbrain (left cerebral peduncle, right substantia nigra, red nucleus and medial lemniscus and central region corresponding to the decussation of the superior cerebellar peduncles), the entire basis pontis and the medulla (posterior region corresponding to the tracts and nuclei gracilis and cuneatus). Atrophy symmetrically involved the middle cerebellar peduncles and peridentate and hemispheric cerebellar WM, whereas the superior and inferior cerebellar peduncels were spared. GM atrophy involved the cerebellar cortex in the superior vermis and flocculonodular lobules. The cerebral aqueduct and the fourth ventricle were not enlarged.

**Figure 2:**
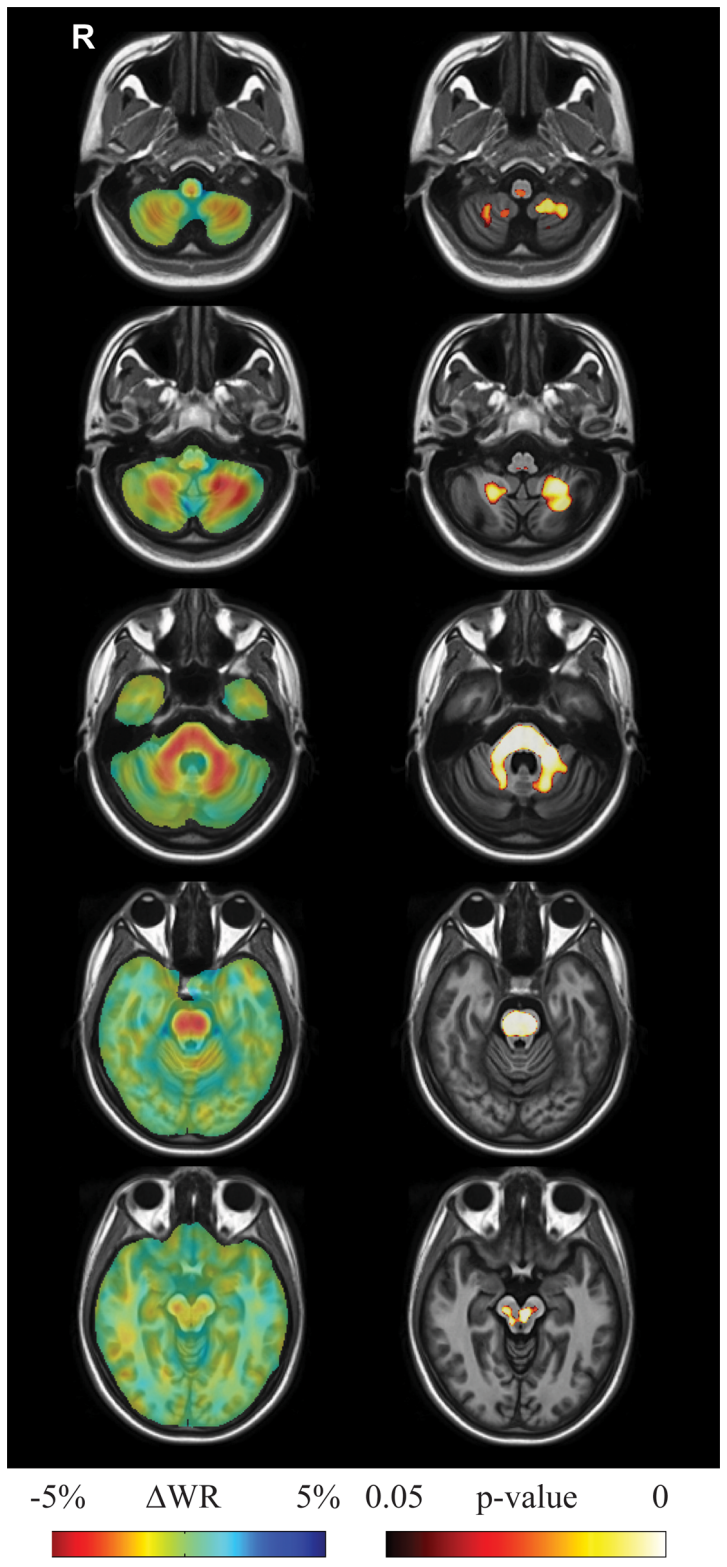
Results of longitudinal between group (SCA2 vs. controls) TBM analysis. Left pane: Sample axial views of the difference in average longitudinal warp rate (ΔWR) maps between SCA2 patients and healthy controls, where red indicates local atrophy and blue indicates local enlargement. Right pane: Voxel-wise corrected p-value maps (threshold-free cluster enhancement, TFCE), testing the null hypothesis of zero differences in WR between SCA2 patients and healthy controls. Highlighted clusters indicate significantly (p<0.05) more pronounced mean atrophy in SCA2 patients when compared to healthy controls (i.e. WR in SCA2 patients significantly lower than WR in control subjects). All maps are overlayed on the population-specific T1 template. SCA2 patients exhibit significant volume loss (higher atrophy rates with respect to controls) in the midbrain (substantia nigra and medial lemniscus, bilaterally, right lateral lemniscus and central region corresponding to decussation of the superior cerebellar peduncles), the entire basis pontis, the middle cerebellar peduncles and posterior medulla corresponding to the in the gracilis and cuneatus tracts and nuclei. The cerebellum shows loss of WM in the hemispheric and peridentate region and of GM in the cerebellar cortex of the inferior portions of the cerebellar hemisphers.

Between-group longitudinal analysis (group x time interaction) (Figure 2, Table S4) showed that SCA2 patients exhibited significantly greater volume loss (higher atrophy rates with respect to controls, i.e. WR values in SCA2 patients were significantly lower than WR values in controls) in the midbrain (substantia nigra and medial lemniscus, bilaterally, right lateral lemniscus and central region corresponding to decussation of the superior cerebellar peduncles), the entire basis pontis, the middle cerebellar peduncles and posterior medulla corresponding to the in the gracilis and cuneatus tracts and nuclei. The cerebellum showed loss of WM in the hemispheric and peridentate region and of the GM in the cerebellar cortex of the inferior portions of the cerebellar hemisphers. No volume change was observed in the supratentorial compartment except for enlargement (i.e. WR values in SCA2 patients were significantly greater than WR values in controls) of the cerebral ventricles.

The mean IACRS score changed from 17.2 at baseline to 21.3 at follow-up (23.8% worsening, p = 0.005, paired t-test). No significant correlation between WR and disease duration, number of abnormal CAG triplets and modification of the neurological deficit between baseline and follow-up MRI was observed.

## Discussion

SCA2 belongs to the group of polyglutamine diseases which include nine neurodegenerative conditions sharing abnormal expansion of a CAG triplet in the coding region of the mutated gene as fundamental pathogenetic mechanism [3]. In particular, SCA2 involves expansion in excess of 32 CAG repeats in the disease gene Ataxin-2, which is inherited as an autosomal dominant tract. The expanded Ataxin-2 mainly targets several pontine neurons and Purkinje cells in the cerebellum, localizes to RNA containing stress granules and associates with the endoplasmic reticulum/Golgi fraction, which is assumed to play a role in cytoplasmic RNA-related functions [2], [39], [40].

Besides cerebellar ataxia, clinical features of SCA2 are variable and early saccadic slowing, pyramidal tract dysfunction, hyporeflexia, myoclonus and severe tremor are commonly observed [1]. The rate of clinical progression of SCA2 is higher with increasing CAG expansion size and decreasing age at onset [2], [15], [41].

Several recent studies focused on defining appropriate clinical measurements of progression of the neurological deficit in SCA2 [2], [41]–[43]. However, all clinical scales are operator-dependent and have limited sensitivity to disease progression [44]. In particular, based on longitudinal clinical examinations spanning 2.4 years, in serially examined SCA2 patients it was estimated that a minimum sample size of 57 was necessary to demonstrate a 50% reduction in clinical progression using a dedicated neurological scale [43].

Gross neuropathological examination of the brain in SCA2 reveals a pattern of pontocerebellar atrophy combined with variable loss of bulk of the inferior olives and widening of the sulci of the frontal lobes [1], [45]–[50]. Decrease in the cerebellum's weight correlated with longer disease durations, confirming the neurodegenerative type of disease evolution [46].

Also, the microscopic neuropathological examination of the central nervous system shows a certain degree of heterogeneity in SCA2. Neuronal loss is widespread but prominent in the cerebellar cortex and pontine nuclei, the midbrain, medulla, and motor cortex. The thalamus, basal ganglia, the remainder of the cerebral cortex and the Clarke column in the spinal cord are affected late in the disease course [1], [45]–[51], however the dentate is relatively spared [46], [48], [50]. WM damage in SCA2 consists of loss of myelinated fibers and gliosis affecting the transverse pontine fibres, central cerebellum and the cerebellar folia, the middle and inferior cerebellar peduncles, medial lemniscus and trigeminal tracts, the fasciculus gracilis and cuneatus and the spinocerebellar tracts [1], [45], [46], [49], [51].

MRI-based studies are able to show loss of bulk of the brainstem and cerebellum, consistent with a pattern of pontocerebellar atrophy, which is already visible in presymptomatic and early symptomatic SCA2 gene carriers [5], [13], [52]. All VBM studies confirmed atrophy of the brainstem, middle cerebellar peduncles and cerebellum in symptomatic SCA2 gene carriers [4], [6]–[8], [10] which, in some studies, was combined with circumscribed atrophy of the frontal, parietal and temporal cortex, thalamus and frontal and temporal white matter [4], [8], [10]. The results of our between-group TBM analysis at baseline confirms the predominant pontocerebellar atrophy pattern in SCA2.

Previous cross-sectional studies reported a variable correlation between GM and WM volume loss and disease duration as well as severity of the clinical deficit [4], [6]–[9], [11], [14]–[16]. In particular, the absence of such correlations was hypothesized to reflect the presence of atrophy before the onset of symptoms and a slower rate of progression of neurodegeneration in SCA2 as compared to SCA1, SCA3 and SCA17 [11], [13], [41], [43].

In contrast with pathological examinations, MRI allows *in vivo*, non-invasive tracking of the progression of structural brain damage in patients with neurodegenerative disease [31], [53], [54]. This capability provides a better understanding of disease physiopathology and suggests using MRI as a biomarker in clinical trials.

In a longitudinal VBM study in nine patients with SCA17 examined twice 18 months apart, on one hand progression of GM atrophy in cerebellar and cerebral motor networks correlated with deterioration of the motor function, and on the other hand atrophic changes in frontal, limbic, parietal and cerebellar regions correlated with severity of psychiatric disturbances [19].

A combined approach using semi-automatic ROI-based volumetry and VBM in patients with SCA1 (n = 37), SCA3 (n = 19) and SCA6 (n = 7) examined 2 years apart showed that progression of brain atrophy particularly affected the brainstem and cerebellum in SCA1, the putamen and pallidum in SCA3, and the cerebellum as well as the thalamus, putamen, caudate and pallidum in SCA6 [18]. CAG repeat length correlated with atrophy progression of the cerebellum and pons in SCA1, whereas no correlation between the progression of atrophy and of the clinical deficit was observed.

Finally, VBM failed to demonstrate progression of brain atrophy after one year in 45 patients with SCA3 as compared to 51 healthy controls [17].

In the present study, we explored longitudinal changes of brain volume in 10 SCA2 patients and 16 age- and gender-matched healthy controls. TBM has proven to be a robust, high-throughput imaging marker in Alzheimer's disease and mild cognitive impairment [22], [24], [25], Parkinson disease [55], [56] and Huntington disease [31], and is particularly well suited for longitudinal studies [24], [31]. To the best of our knowledge, so far TBM has never been employed to assess atrophy distribution and progression in inherited or sporadic ataxias.

In our SCA2 patients, between-group longitudinal TBM analysis showed progression of atrophy of the brainstem, middle cerebellar peduncles and cerebellum during the follow-up period. This result indicates that neurodegeneration in SCA2 is associated with an ongoing pontocerebellar atrophy process. Notably, this aspect *per se* is not incompatible with demonstration of brainstem and cerebellar volume loss already present in presymptomatic SCA2 gene carriers [13], [52], which was tentatively attributed to a possible developmental (rather than degenerative) component of SCA2 physiopathology [14].

The same analysis showed additional progressive volume loss in the midbrain, which partly corresponded to the substantia nigra and medial lemnisci, and in the medulla, which corresponded to the gracilis and cuneatus tracts and nuclei. Involvement of the substantia nigra in SCA2 was consistently reported in neuropathological studies [39] and is in agreement with the common occurrence of parkinsonism or subclinical nigrostriatal dysfunction [1], [57], [58]. In turn, the involvement of cuneatus and gracilis tracts and nuclei and medial lemnisci is recognized pathologically in SCA2 [39], [51] and in line with clinical symptoms and signs of somatosensory involvement in SCA2 [51].

We also explored the correlation between the rate of progression of volume changes and clinical deficit. The lack of correlation between atrophy rate and clinical deterioration which we observed in SCA2 is in line with the results obtained in SCA1, SCA3 and SCA6, albeit difficult to interpret due to our small sample size.

We recognize two main limitations of the present study. First, we examined a small number of patients. Hence we may have been unable to reveal minor effects, for instance progressive atrophy of the supratentorial brain or correlation between progression of regional atrophy and clinical modifications – such effects could be detectable in a larger cohort. However, with the perspective of translating our results to the design of clinical trials including MRI volumetry in conjunction with TBM as surrogate biomarker, it is important to have been able to measure meaningful local volume differences in a relatively small cohort. Second, we performed a single center study. Implementation of MRI as biomarker for rare diseases such as SCA2 would greatly benefit from access to large-scale, multicenter studies.

## Conclusion

MRI volumetry using TBM is capable of demonstrating the progression of pontocerebellar atrophy in SCA2, supporting a possible role of MRI as biomarker in future trials.

## Supporting Information

Figure S1 **Results of the longitudinal within group (controls) TBM analysis.** Left pane: Sample axial views of average warp rate (WR) maps in healthy controls, where red indicates local atrophy and blue indicates local enlargement. Right pane: voxel-wise corrected p-value maps (threshold-free cluster enhancement, TFCE) testing the null hypothesis of zero WR. Highlighted clusters indicate significant (p<0.05) atrophic changes (i.e. WR significantly lower than zero). All maps are overlayed on the population-specific T1 template. Healthy controls show a circumscribed atrophy of the right frontal mid-orbital gyrus and of the underlying WM.
(TIF)

Figure S2 **Results of the longitudinal within group (SCA2) TBM analysis.** Left pane: Sample axial views of average warp rate (WR) maps in SCA2 patients, where red indicates local atrophy and blue indicates local enlargement. Right pane: voxel-wise corrected p-value maps (threshold-free cluster enhancement, TFCE) testing the null hypothesis of zero WR. Highlighted clusters indicate significant (p<0.05) atrophic changes (i.e. WR significantly lower than zero). All maps are overlayed on the population-specific T1 template. The supratentorial compartement shows WM volume loss in the left frontal gyrus, temporal lobe, anterior limb of the internal capsule, and peritrigonal region, GM volume loss in the left midfrontal gyrus, putamen and pallidus and in the thalamus and hippocampus, bilaterally. In the infratentorial compartment, diffuse volume loss is present in the midbrain (left cerebral peduncle, right substantia nigra, red nucleus and medial lemniscus and central region corresponding to the decussation of the superior cerebellar peduncles), and the entire basis pontis and medulla (posterior region corresponding to the tracts and nuclei gracilis and cuneatus). Atrophy symmetrically involves the middle cerebellar peduncles and peridentate and hemispheric cerebellar WM. GM atrophy involved the cerebellar cortex in the superior vermis and flocculonodular lobules.
(TIF)

Table S1 **Results of the baseline between group (SCA2 vs. controls) TBM analysis.** p-values and MNI coordinates (Talairach Daemon Labels) of local extrema within clusters of significantly (p<0.05, threshold-free cluster enhancement, TFCE) more pronounced mean atrophy in SCA2 patients when compared to healthy controls (i.e. **|J|**_baseline_ in SCA2 patients significantly lower than **|J|**_baseline_ in healthy controls).
(DOC)

Table S2 **Results of the longitudinal within group (controls) TBM analysis.** p-values and MNI coordinates (Talairach Daemon Labels) of local extrema within clusters of significantly (p<0.05, threshold-free cluster enhancement, TFCE) mean atrophy in healthy controls (i.e. Warp Rate (WR) significantly lower than zero).
(DOC)

Table S3 **Results of the longitudinal within group (SCA2) TBM analysis.** p-values and MNI coordinates (Talairach Daemon Labels) of local extrema within clusters of significantly (p<0.05, threshold-free cluster enhancement, TFCE) mean atrophy in SCA2 patients (i.e. Warp Rate (WR) significantly lower than zero).
(DOC)

Table S4 **Results of longitudinal between group (SCA2 vs. controls) TBM analysis.** p-values and MNI coordinates (Talairach Daemon Labels) of local extrema within clusters of significantly (p<0.05, threshold-free cluster enhancement, TFCE) more pronounced mean atrophy in SCA2 patients when compared to healthy controls (i.e. Warp Rate (WR) in SCA2 patients significantly lower than Warp Rate (WR) in control subjects).
(DOC)
