# Supplementary figures and images for: Progression of Brain Atrophy in Spinocerebellar Ataxia Type 2: A Longitudinal Tensor-Based Morphometry Study
Source: PLoS One. 2014 Feb 25;9(2):e89410. doi: 10.1371/journal.pone.0089410 (PMC3934889; doi:10.1371/journal.pone.0089410)

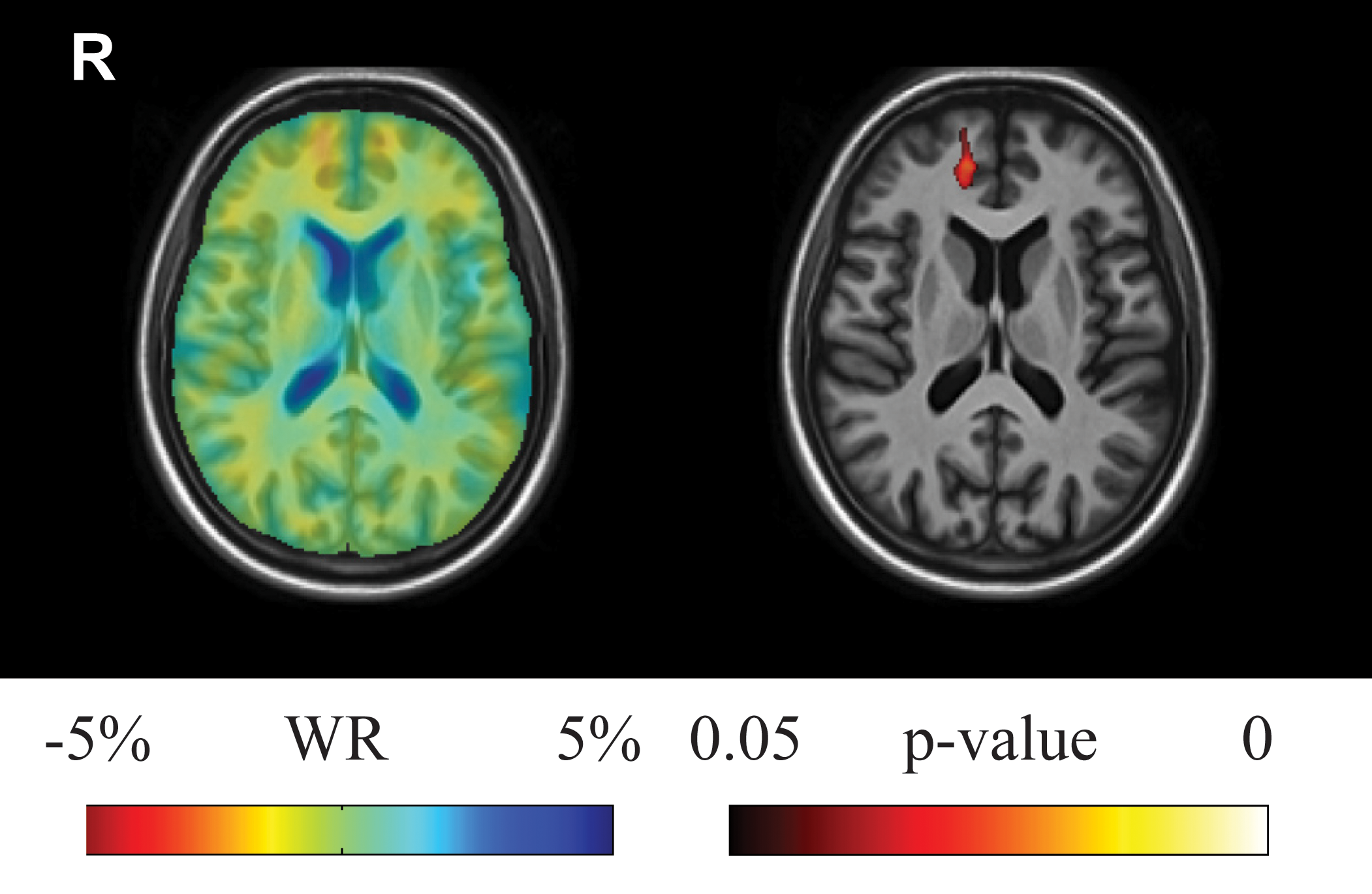

Supplement: Figure S1 — Results of the longitudinal within group (controls) TBM analysis. Left pane: Sample axial views of average warp rate (WR) maps in healthy controls, where red indicates local atrophy and blue indicates local enlargement. Right pane: voxel-wise corrected p-value maps (threshold-free cluster enhancement, TFCE) testing the null hypothesis of zero WR. Highlighted clusters indicate significant (p<0.05) atrophic changes (i.e. WR significantly lower than zero). All maps are overlayed on the population-specific T1 template. Healthy controls show a circumscribed atrophy of the right frontal mid-orbital gyrus and of the underlying WM. (TIF) [file pone.0089410.s001.tif]

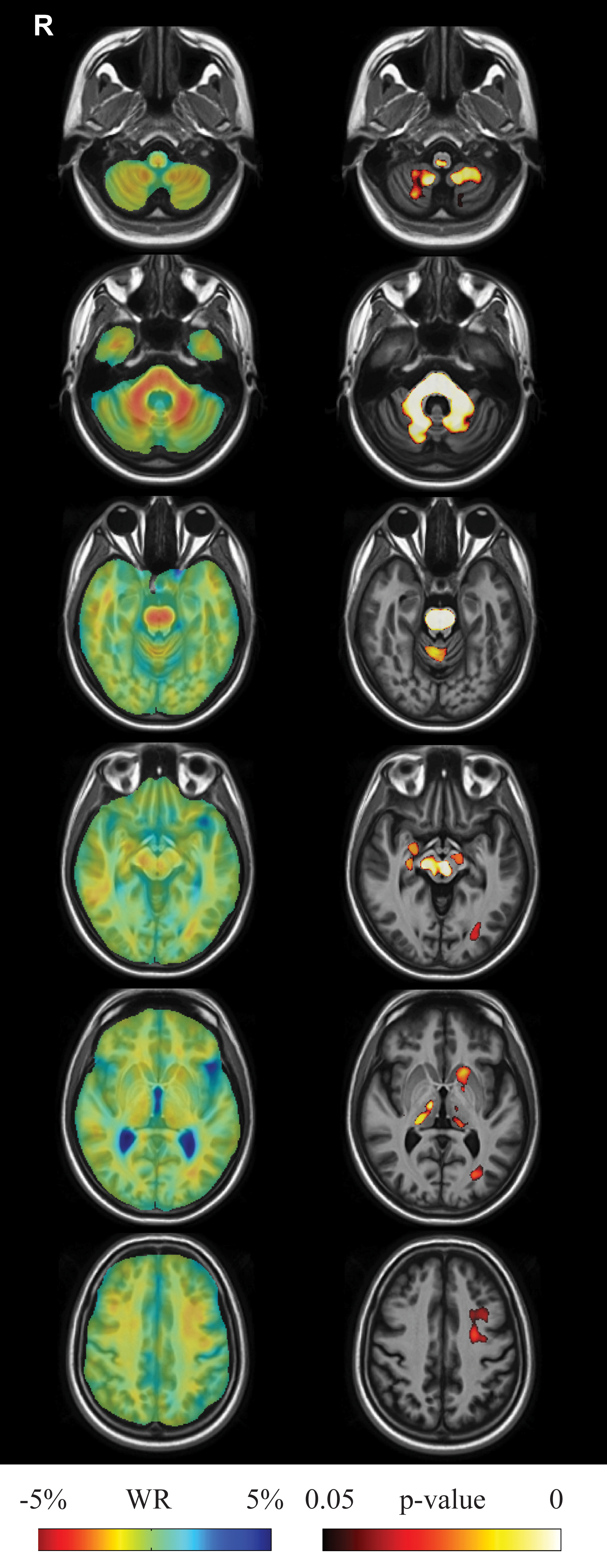

Supplement: Figure S2 — Results of the longitudinal within group (SCA2) TBM analysis. Left pane: Sample axial views of average warp rate (WR) maps in SCA2 patients, where red indicates local atrophy and blue indicates local enlargement. Right pane: voxel-wise corrected p-value maps (threshold-free cluster enhancement, TFCE) testing the null hypothesis of zero WR. Highlighted clusters indicate significant (p<0.05) atrophic changes (i.e. WR significantly lower than zero). All maps are overlayed on the population-specific T1 template. The supratentorial compartement shows WM volume loss in the left frontal gyrus, temporal lobe, anterior limb of the internal capsule, and peritrigonal region, GM volume loss in the left midfrontal gyrus, putamen and pallidus and in the thalamus and hippocampus, bilaterally. In the infratentorial compartment, diffuse volume loss is present in the midbrain (left cerebral peduncle, right substantia nigra, red nucleus and medial lemniscus and central region corresponding to the decussation of the superior cerebellar peduncles), and the entire basis pontis and medulla (posterior region corresponding to the tracts and nuclei gracilis and cuneatus). Atrophy symmetrically involves the middle cerebellar peduncles and peridentate and hemispheric cerebellar WM. GM atrophy involved the cerebellar cortex in the superior vermis and flocculonodular lobules. (TIF) [file pone.0089410.s002.tif]
