# Supplementary material for: Progression of Brain Atrophy in Spinocerebellar Ataxia Type 2: A Longitudinal Tensor-Based Morphometry Study
Source: PLoS One. 2014 Feb 25;9(2):e89410. doi: 10.1371/journal.pone.0089410 (PMC3934889; doi:10.1371/journal.pone.0089410)
Supplement: Table S1 — Results of the baseline between group (SCA2 vs. controls) TBM analysis. p-values and MNI coordinates (Talairach Daemon Labels) of local extrema within clusters of significantly (p<0.05, threshold-free cluster enhancement, TFCE) more pronounced mean atrophy in SCA2 patients when compared to healthy controls (i.e. |J| baseline in SCA2 patients significantly lower than |J| baseline in healthy controls). (DOC) [file pone.0089410.s003.doc]

**Table S1.** **Results of the baseline between group (SCA2 vs. controls) TBM analysis.** p-values and MNI coordinates (Talairach Daemon Labels) of local extrema within clusters of significantly (p<0.05, threshold-free cluster enhancement, TFCE) more pronounced mean atrophy in SCA2 patients when compared to healthy controls (i.e. **|J|**_baseline_ in SCA2 patients significantly lower than **|J|**_baseline_ in healthy controls).

| Talairach Daemon Labels area | p value | X  (mm) | Y  (mm) | Z  (mm) |
| --- | --- | --- | --- | --- |
| Left Cerebellum.Anterior Lobe.*.Gray Matter.* | 0.001 | -8 | -45 | -27 |
| Left Cerebellum.Anterior Lobe.Nodule.Gray Matter.* | 0.001 | 0 | -63 | -30 |
| Left Cerebellum.Anterior Lobe.*.Gray Matter.* | 0.001 | -9 | -49 | -31 |
| Left Cerebellum.Anterior Lobe.Nodule.Gray Matter.* | 0.001 | -1 | -60 | -32 |
| Left Cerebellum.Posterior Lobe.Cerebellar Tonsil.Gray Matter.* | 0.001 | -4 | -61 | -33 |
| Left Cerebellum.Posterior Lobe.Uvula.Gray Matter.* | 0.001 | -8 | -65 | -33 |

L, left; R, right. Coordinates are expressed in MNI standard space.
