# Supplementary material for: Progression of Brain Atrophy in Spinocerebellar Ataxia Type 2: A Longitudinal Tensor-Based Morphometry Study
Source: PLoS One. 2014 Feb 25;9(2):e89410. doi: 10.1371/journal.pone.0089410 (PMC3934889; doi:10.1371/journal.pone.0089410)
Supplement: Table S2 — Results of the longitudinal within group (controls) TBM analysis. p-values and MNI coordinates (Talairach Daemon Labels) of local extrema within clusters of significantly (p<0.05, threshold-free cluster enhancement, TFCE) mean atrophy in healthy controls (i.e. Warp Rate (WR) significantly lower than zero). (DOC) [file pone.0089410.s004.doc]

**Table S2. Results of the longitudinal within group (controls) TBM analysis.** p-values and MNI coordinates (Talairach Daemon Labels) of local extrema within clusters of significantly (p<0.05, threshold-free cluster enhancement, TFCE) mean atrophy in healthy controls (i.e. Warp Rate (WR) significantly lower than zero).

| Talairach Daemon Labels area | p value | X  (mm) | Y  (mm) | Z  (mm) |
| --- | --- | --- | --- | --- |
| Right Cerebrum.Frontal Lobe.Medial Frontal Gyrus.Gray Matter.Brodmann area 10 | 0.024 | 13 | 48 | -4 |
| Right Cerebrum.Limbic Lobe.Anterior Cingulate.White Matter.* | 0.024 | 12 | 46 | -4 |
| Right Cerebrum.Frontal Lobe.Medial Frontal Gyrus.White Matter.* | 0.041 | 14 | 63 | -4 |

L, left; R, right. Coordinates are expressed in MNI standard space.
