# Supplementary material for: Progression of Brain Atrophy in Spinocerebellar Ataxia Type 2: A Longitudinal Tensor-Based Morphometry Study
Source: PLoS One. 2014 Feb 25;9(2):e89410. doi: 10.1371/journal.pone.0089410 (PMC3934889; doi:10.1371/journal.pone.0089410)
Supplement: Table S3 — Results of the longitudinal within group (SCA2) TBM analysis. p-values and MNI coordinates (Talairach Daemon Labels) of local extrema within clusters of significantly (p<0.05, threshold-free cluster enhancement, TFCE) mean atrophy in SCA2 patients (i.e. Warp Rate (WR) significantly lower than zero). (DOC) [file pone.0089410.s005.doc]

**Table S3. Results of the longitudinal within group (SCA2) TBM analysis**. p-values and MNI coordinates (Talairach Daemon Labels) of local extrema within clusters of significantly (p<0.05, threshold-free cluster enhancement, TFCE) mean atrophy in SCA2 patients (i.e. Warp Rate (WR) significantly lower than zero).

| Talairach Daemon Labels area | p value | X  (mm) | Y  (mm) | Z  (mm) |
| --- | --- | --- | --- | --- |
| Right Brainstem.Midbrain.*.*.* | 0.001 | 8 | -31 | -18 |
| Right Brainstem.Pons.*.*.* | 0.001 | 1 | -26 | -23 |
| Left Brainstem.Pons.*.*.* | 0.001 | 0 | -29 | -23 |
| Right Brainstem.Pons.*.*.* | 0.001 | 8 | -24 | -35 |
| Left Brainstem.Pons.*.*.* | 0.001 | -3 | -22 | -36 |
| Left Brainstem.Pons.*.*.* | 0.001 | -6 | -23 | -36 |
| Left Cerebrum.Frontal Lobe.Sub-Gyral.White Matter.* | 0.029 | -25 | 3 | 38 |
| Left Cerebrum.Frontal Lobe.Sub-Gyral.White Matter.* | 0.036 | -34 | -1 | 39 |
| Left Cerebrum.Frontal Lobe.Precentral Gyrus.White Matter.* | 0.037 | -36 | 19 | 37 |
| Left Cerebrum.Frontal Lobe.Precentral Gyrus.White Matter.* | 0.037 | -36 | 20 | 35 |
| Left Cerebrum.Frontal Lobe.Sub-Gyral.White Matter.* | 0.038 | -30 | 22 | 26 |
| Left Cerebrum.Frontal Lobe.Sub-Gyral.White Matter.* | 0.041 | -24 | 19 | 33 |
| Left Cerebrum.Occipital Lobe.Middle Occipital Gyrus.White Matter.* | 0.026 | -27 | -73 | 12 |
| Left Cerebrum.Occipital Lobe.Cuneus.White Matter.* | 0.027 | -25 | -73 | 13 |
| Left Cerebrum.Occipital Lobe.Middle Occipital Gyrus.White Matter.* | 0.027 | -25 | -75 | 12 |
| Left Cerebrum.Occipital Lobe.Sub-Gyral.White Matter.* | 0.03 | -30 | -73 | 3 |
| Left Cerebrum.Occipital Lobe.Middle Occipital Gyrus.White Matter.* | 0.031 | -30 | -79 | 7 |
| Left Cerebrum.Occipital Lobe.Middle Occipital Gyrus.White Matter.* | 0.031 | -31 | -77 | 6 |
| Left Cerebrum.Temporal Lobe.Sub-Gyral.White Matter.* | 0.048 | -35 | -54 | 23 |
| Left Cerebrum.Temporal Lobe.Superior Temporal Gyrus.White Matter.* | 0.05 | -38 | -52 | 20 |

L, left; R, right. Coordinates are expressed in MNI standard space.
