# Supplementary material for: Progression of Brain Atrophy in Spinocerebellar Ataxia Type 2: A Longitudinal Tensor-Based Morphometry Study
Source: PLoS One. 2014 Feb 25;9(2):e89410. doi: 10.1371/journal.pone.0089410 (PMC3934889; doi:10.1371/journal.pone.0089410)
Supplement: Table S4 — Results of longitudinal between group (SCA2 vs. controls) TBM analysis. p-values and MNI coordinates (Talairach Daemon Labels) of local extrema within clusters of significantly (p<0.05, threshold-free cluster enhancement, TFCE) more pronounced mean atrophy in SCA2 patients when compared to healthy controls (i.e. Warp Rate (WR) in SCA2 patients significantly lower than Warp Rate (WR) in control subjects). (DOC) [file pone.0089410.s006.doc]

**Table S4.** **Results of longitudinal between group (SCA2 vs. controls) TBM analysis.** p-values and MNI coordinates (Talairach Daemon Labels) of local extrema within clusters of significantly (p<0.05, threshold-free cluster enhancement, TFCE) more pronounced mean atrophy in SCA2 patients when compared to healthy controls (i.e. Warp Rate (WR) in SCA2 patients significantly lower than Warp Rate (WR) in control subjects).

| Talairach Daemon Labels area | p value | X  (mm) | Y  (mm) | Z  (mm) |
| --- | --- | --- | --- | --- |
| Left Brainstem.Midbrain.*.*.* | 0.001 | -2 | -27 | -13 |
| Left Brainstem.Midbrain.*.*.* | 0.001 | -1 | -25 | -22 |
| Left Brainstem.Pons.*.*.* | 0.001 | 0 | -31 | -24 |
| Left Brainstem.Pons.*.*.* | 0.001 | -5 | -21 | -34 |
| Left Brainstem.Pons.*.*.* | 0.001 | -12 | -40 | -34 |
| Left Brainstem.Pons.*.*.* | 0.001 | -3 | -21 | -35 |

L, left; R, right. Coordinates are expressed in MNI standard space.
